# SIMPLE SOLUTIONS TO A COMPLEX PROBLEM: LISTENING TO DIRECT CARE WORKERS’ PERCEPTIONS ABOUT RETENTION

**DOI:** 10.1093/geroni/igac059.3141

**Published:** 2022-12-20

**Authors:** Isha Karmacharya, Leah Janssen

**Affiliations:** Miami University, Oxford, Ohio, United States; Miami University, Oxford, Ohio, United States

## Abstract

Direct care workers (DCWs) play a crucial role in delivering long-term services in nursing homes, assisted living, and in-home care. The work is physically and emotionally demanding and generally low-paid, which makes retaining and managing the workforce extremely challenging and has resulted in an unending workforce crisis. This study provides data on retention-promoting factors from the perspective of DCWs working in high-performing facilities and home care agencies that were identified through national and state retention data and reviews from case management agencies. Qualitative interviews were conducted in the spring of 2022 with a purposive sample of DCWs (n=16). Interviews were transcribed, checked for accuracy, and coded via thematic analysis. Three overarching themes emerged: work culture, appreciation, and monetary benefits. Retention was supported by a work culture that emphasizes person-centered care, family atmosphere, relationship building, staff empowerment, coaching supervision, participative leadership, effective communication, and flexible working conditions. DCWs experienced appreciation from employers, supervisors, and clients, which they perceived to promote retention. This theme includes valuing, respecting, recognizing, and acknowledging the efforts of DCWs. Monetary benefits promoting retention were seen as regular wage raises, employee assistance funds, gift cards, bonuses, paid time off, health insurance benefits, and travel reimbursement. This study demonstrates that while worker retention is a crisis, there are modifiable aspects of the job that can promote workers’ retention. DCWs’ retention can be impacted by work culture and appreciation practices. Study findings have implications for facility policy and practice pertaining to DCW’s retention.

